# TOWARD, a metabolic health intervention, demonstrates robust 1-year weight loss and cost-savings through deprescription

**DOI:** 10.3389/fnut.2025.1548609

**Published:** 2025-02-14

**Authors:** Laura Buchanan, Matthew Calkins, Tro Kalayjian, Nicholas G. Norwitz, Nina Teicholz, David Unwin, Adrian Soto-Mota

**Affiliations:** ^1^Private Practice, Tappan, NY, United States; ^2^Atrium Health One Health, Rural Hall, NC, United States; ^3^Harvard Medical School, Boston, MA, United States; ^4^Freelance Journalist, New York, NY, United States; ^5^Faculty of Health Social Care and Medicine, Edge Hill University, Ormskirk, United Kingdom; ^6^Need for Nutrition Education Project (NNEdPro) Global Institute for Food Nutrition and Health, Cambridge, United Kingdom; ^7^Metabolic Diseases Research Unit, National Institute of Medical Sciences and Nutrition Salvador Zubiran, Mexico City, Mexico; ^8^Tecnologico de Monterrey, School of Medicine, Mexico City, Mexico

**Keywords:** GLP1, diabetes, ketogenic, obesity, telemedicine, therapeutic carbohydrate reduction, carnivore diet, intermittent fasting

## Abstract

**Background:**

Cost, scalability, and durability represent major challenges to the implementation of intensive lifestyle treatments for obesity and diabetes. We previously reported pilot data from a 6-month intervention in which a self-insured manufacturing company partnered with a metabolic health clinic that utilizes therapeutic carbohydrate reduction (TCR), asynchronous monitoring, and a community-based approach to treat employees with metabolic disease. This manuscript presents weight loss and cost-savings from deprescription at the 12-month time point.

**Methods:**

50 employees, mean BMI 43.2 ± 8.7 kg/m^2^, 64% with prediabetes or type 2 diabetes, were enrolled in the multimodal TOWARD telemedicine intervention, which includes: Text-based communications, Online interactions, Wellness coaching, Asynchronous education, Real-time biofeedback and remote monitoring, and Dietary modifications that emphasizes TCR.

**Results:**

41 completed the one-year intervention. Mean weight loss for the 50 subjects in the intention-to-treat analysis was 19.5 ± 11.4 kg, corresponding to 15.5% total body weight loss with concomitant deprescription of 96 medications, while starting only 8 medications. In patients who discontinued GLP-1 receptor agonists, weight loss continued or was maintained. Annualized cost savings from the TOWARD approach were approximately -$1700 per patient, as compared to an annualized cost burden of roughly +$13000 per patient for a GLP-1 receptor agonist.

**Conclusion:**

The TOWARD approach represents a scalable metabolic health intervention that demonstrates robust improvements in weight while simultaneously allowing for deprescription leading to substantial cost savings. TOWARD could serve as a scalable tool to facilitate intensive lifestyle intervention with efficacy on par with GLP-1 receptor agonists.

## Graphical Abstract

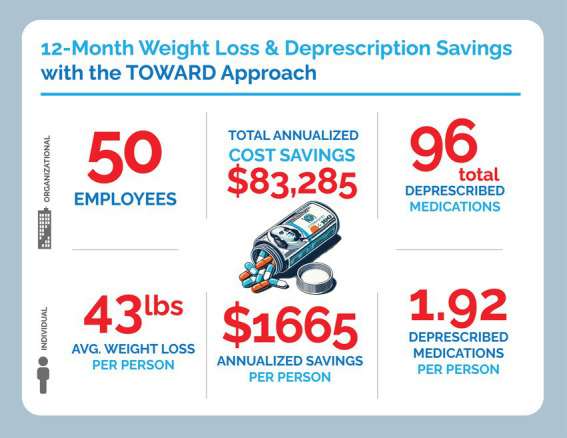

## Introduction

Behavioral and lifestyle changes are considered a viable first-line treatment for diet-related noncommunicable diseases, including Type 2 Diabetes (T2D), prediabetes, hypertension, metabolic syndrome and obesity (1). The prevalence of these diseases is increasing, with 38% of adults in the United States having prediabetes or diabetes and 42% having obesity (2, 3). The economic burden of metabolic disease is also high, with $412 billion spent on T2D and $173 billion on obesity annually in the form of direct care cost (4).

Novel medications, including GLP-1 receptor agonists (GLP-1 RA), are becoming a cornerstone of obesity management of metabolic disease given demonstrated improvements in glycemia, adiposity, and cardiovascular outcomes (5). However, these medications are not without limitations. Trials have consistently found weight regain upon discontinuation (6), necessitating a lifetime commitment to the drug to retain weight loss and other health benefits. Some patients are nonresponders and do not meet the clinically significant weight loss goal of 5%. Fully 25% of subjects in the semaglutide 2.4 mg “STEP 5” trial experienced a < 5% reduction in body weight over 104 weeks (6). This trial also reported that cravings and hunger started to return toward baseline at 20 weeks to nearly or entirely equal to the placebo group at week 104 (6). An analysis of the SUSTAIN 8 clinical trial found that 30 to 40% of weight loss came from lean body mass (muscle) with the use of semaglutide over 52 weeks (7). Lean mass loss with GLP-1RA is of potential long-term concern because of the implications of sarcopenia on metabolism and its association with increased all-cause mortality (8).

Serious and widespread side effects of GLP-1 RAs are additional cause for concern. Some 82.2% of subjects in the STEP 5 trial were reported to experience gastrointestinal side effects on semaglutide 2.4 mg at 2 years (5). A small but notable increase in risk for all thyroid cancer and medullary thyroid cancer was also observed after 1–3 years treatment on GLP-1RAs in an analysis from the French national health care insurance system database (9).

For people with diabetes, outcomes may be less robust than reported. For instance, a follow-up study to the SCOPE trial (semaglutide 2.4 mg) reported significantly reduced HbA1c, yet this observation was derived from data on only 30 out of 343 subjects (10). Further, in a real-world retrospective study of 82,624 adults (mostly men), GLP-1RAs were found to be the least well tolerated medication, with a deprescription rate of more than 50%, among 5 medications prescribed for people with T2D. This deprescription rate was 10% higher than the next-most deprescribed drug (11). Another real-world study published in July 2023 investigated claims of 16 million commercially insured members and found that almost 70% of patients stopped GLP-1RA treatment within less than a year after starting (12). A prominent concern with these weight loss therapeutics is the price tag, with more than 142 million Americans qualifying for the medication and a price ranging from $800 to $1700/month, annual costs on the healthcare system could reach over a trillion dollars (13). The need for cost-effective alternatives as either a replacement or adjunct to GLP-1 RAs is therefore paramount.

Two studies have documented sustained weight loss maintenance with GLP-1RA discontinuations. A supervised exercise intervention was found to maintain body weight and body composition for 1 year following the discontinuation of GLP-1RA (14). Likewise, a retrospective analysis on 154 individuals demonstrated that a ketogenic diet prevented weight regain and protected against any increase in HbA1c at both six and 12 months following deprescription from GLP-1 RAs (15). The mean HbA1c of the deprescribed cohort rose 0.2% at 12 months yet remained in the non-diabetic range (15). These data suggest that programs that incorporate intensive health coaching, exercise prescriptions and TCR may represent important interventions to mitigate cost as well as limit weight regain and potential other harms from GLP-1 RA use.

Historically, few employee wellness programs have documented strong efficacy in managing diabetes or obesity. Our previously published 6-month data using the TOWARD approach demonstrated significant results on weight, Atherosclerotic Cardiovascular Disease (ASCVD) risk and cost-savings (16). In this paper, we present a focused review of the one-year weight loss results and cost savings from medication deprescription from 50 patients who self-selected to participate.

## Materials and methods

### Intervention

**Figure 1:**
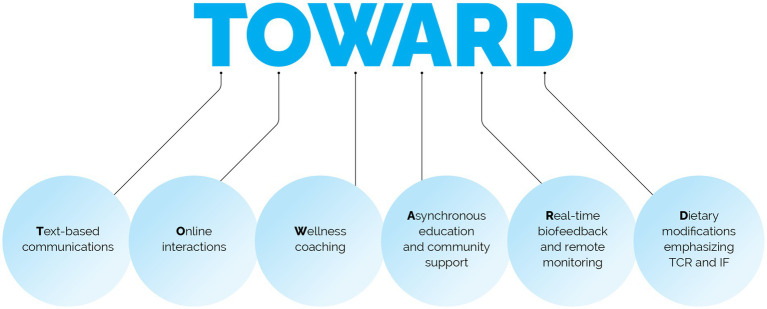
Components of the intervention.

The TOWARD intervention employed by the metabolic health clinic is a combination of five well-defined, evidence-based best practices, including Text-based communications and messaging, Online interactions, Wellness coaching, Asynchronous education & community support, Real-time biofeedback and remote monitoring of body composition, blood pressure, blood glucose and ketones, as well as Dietary modifications that emphasize TCR and intermittent fasting (IF) approaches (Figure 1).

More detailed information about each component as follows:

(1) Text-Messaging. HIPAA-compliant texting apps have been shown to be a feasible method of increasing patient engagement in treatment, improving outcomes, and monitoring patients’ treatment progress over time (17, 18). Literature suggests that mobile interactions can decrease HbA1c in patients with diabetes (19). Patients who enrolled in the employee wellness program were encouraged to use the clinic’s smartphone app and direct messaging platform to facilitate behavior change and ask questions of their health care teams.
(2) Online interactions with Wellness coaches and clinical teams. Telemedicine programs for weight loss have flourished over the past several years, with robust data demonstrating weight loss from various approaches, including general wellness approaches that promote caloric restriction and increased exercise (20). More notably, telemedicine programs that specifically encourage TCR have demonstrated robust outcomes for T2D remission (21).

- Telemedicine programs that also address patient-food relationship and utilize health coaches have demonstrated superior weight loss and diabetes results (21, 22). The care team for this metabolic health management pilot program included two physicians, one physician assistant, three medical assistants, four health coaches and one personal trainer. Patients enrolled online and completed application onboarding which included review of prior diets, medications, mental health and eating disorders, and readiness to change and overall wellness to enable some personalization of care. Leveraging telemedicine capability, patients initially met with a medical assistant online to receive an overview of the program, assist with the implementation of their remote monitoring equipment, and begin using the metabolic clinic’s smartphone application. Health Coaches would meet independently with the patient, providing them with educational and behavioral assignments. Initial medical appointments would review a patient’s prior lab results, medical history, personal goals for the program, and food restrictions and preferences. Thereafter, patients attended weekly virtual meetings with rotating members of their care team. Between weekly visits with a member of the care team patients interacted with their care team in individual and group telemedicine visits, via text, email, or secure messaging.

(3) Asynchronous education & community support. Asynchronous education platforms and care models which focus on patient education outside of traditional medical visits have demonstrated improved outcomes in diabetes, hypertension, and obesity (23). Likewise, group meetings both live and via telemedicine have demonstrated improvements in glycemia and food addiction symptoms (23). All employees were given access to the clinic’s smartphone application that included a self-guided multi-media curriculum focusing on the science of hunger, appetite, food addiction, and cravings, as well as the role of stress and sleep hygiene on health and well-being, as well as a monitored community group chat and webinars devoted to the company’s employees.
(4) Real-time biofeedback and remote monitoring of body composition, blood pressure, blood glucose and ketones. Studies have demonstrated that remote patient monitoring can lower blood pressure, improve weight loss maintenance, and improve glycemia in subjects with T2D (24). At the start of the program, all patients were prescribed 1 month of CGM use and given a remote scale and blood pressure cuff. After the first month, patients could choose to continue CGM use, transition to periodic finger-stick ketone and glucose measurements, or focus solely on remote weight and often blood pressure monitoring. Patients on multiple diabetes medications were encouraged to continue using CGMs for close monitoring while titrating off their medications. Patients who did choose to use the ketone and glucose meter often used them 1-2x per week. This approach balanced effective biofeedback with cost-effectiveness. In this study, patients were provided Freestyle Libre 3 continuous glucose monitors (CGMs) (Abbott, Abbott Park, Chicago, IL, USA), glucose–ketone meters (Keto Mojo, Nappa, CA, USA), body weight scales, and sphygmomanometers (Qardio, San Francisco, CA, USA). Patients had access to real-time biofeedback of glycemia, ketone measurements, body weight, lean mass, fat mass, water weight and blood pressure. The equipment also allowed for remote monitoring of real-time patient data, facilitating the safe clinical execution of deprescription.
(5) Dietary modifications that emphasize TCR. The American Diabetes Association and the American Heart Association endorse the benefits of TCR for T2D (25), and the Society of Metabolic Health Practitioners endorse the benefits of TCR for the treatment of T2D, obesity, hypertension, and metabolic syndrome (26). Patients in this 50-patient cohort were all initially recommended some variation of a ketogenic diet. The specific dietary composition was designed individually based on the patient’s allergies, food aversions, cravings and taste preferences. However, all patients were instructed to reduce total carbohydrates to fewer than 30 g (advised to not count carbohydrates from non-starchy vegetables or leafy greens) to maintain nutritional ketosis; in this context, patients were also encouraged to eat according to their subjective hunger and experiment with intermittent fasting patterns. All patients were educated on both the potential health benefits and risk of intermittent fasting. If patients agreed to adopt it, they were encouraged to track fasting hours and share this information with their health coach. Some patients ate in an 8-h window, others opted for a 6-h window, while others chose to eat one meal per day. This was at the discretion of the patient and was not systematically monitored outside of patient discussions during their visits. Holistically, the aim of the program was to empower patients with information about how to reach their health goals and then nurture their personal growth with access to self-monitoring tools and a supportive healthcare community.

### Baseline and quarterly assessments

**Figure 2:**
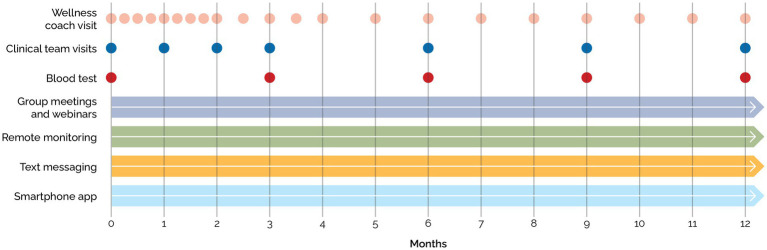
Measurements across time.

Blood tests for all patients at baseline, 12 weeks, 24 weeks, and 52 weeks were collected after a 10–16 h overnight fast and included a complete blood count, comprehensive metabolic panel, thyroid-stimulating hormone, vitamin B12, folate, C-reactive protein, HbA1c, lipid panel, and insulin (Figure 2).

### Establishment of an employee metabolic health wellness program

A self-insured manufacturing company approached the metabolic clinic to establish an employee metabolic wellness program in October 2021. The company advertised the program through internal posters, fliers, emails, and live webinars. The clinic held monthly virtual information sessions for the company. Further outreach was conducted through email campaigns which targeted employees with markers of metabolic syndrome, obesity or diabetes with data obtained from annual corporate lab screenings. Thereafter, employees voluntarily applied to the program, were selected (described below), and established care with the clinic.

### Patient enrollment, consent, and completion

**Figure 3:**
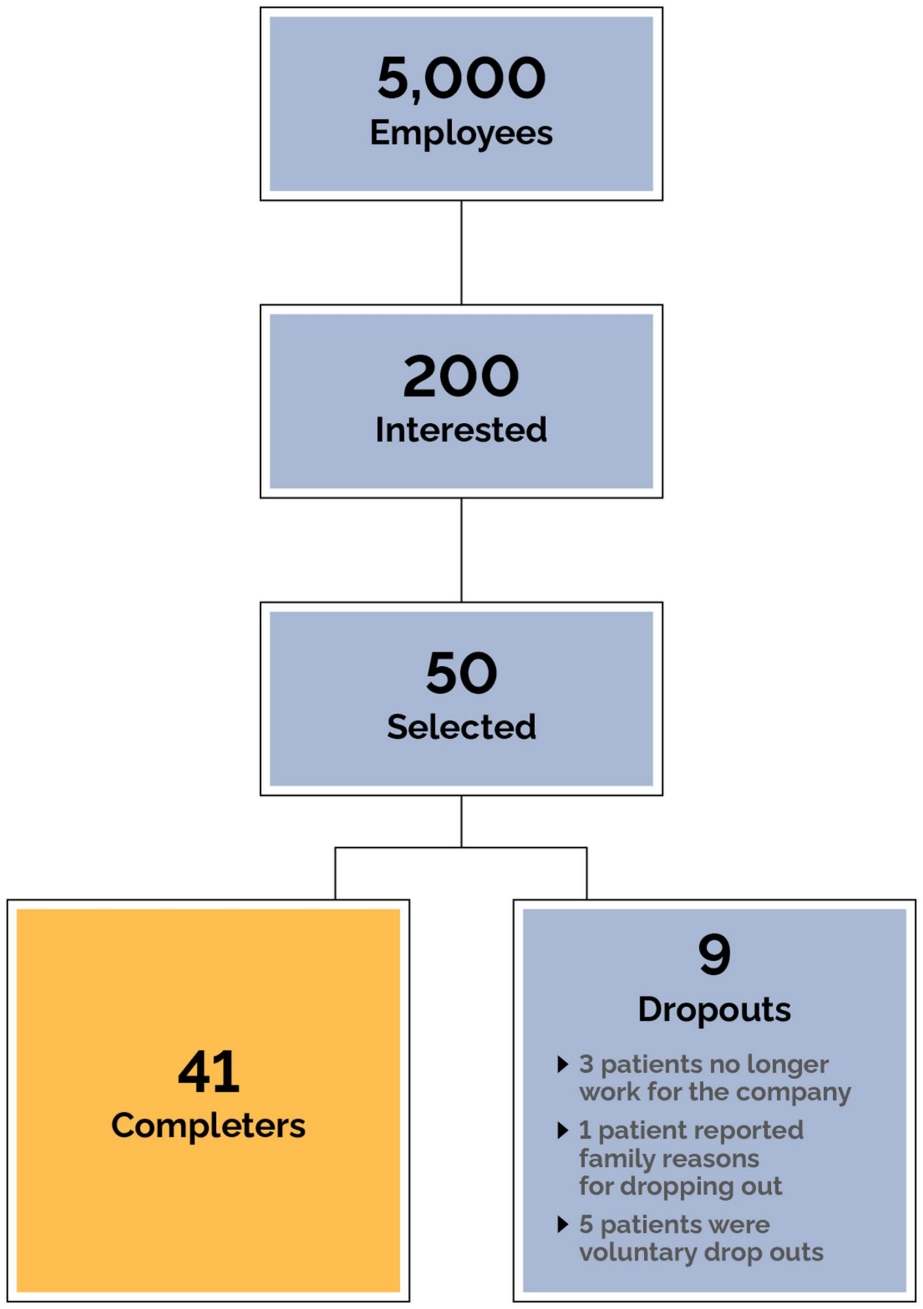
Participant flowchart.

All employees of the company were offered virtual webinars and meetings to learn about metabolic health. After these meetings a QR code was shared with employees to apply if they were interested in participating in the program. An informational campaign to enroll employees using email, posters, and table tents also took place. The clinic received hundreds of applications (Figure 3), and due to limitations in on-boarding capacity, patients were prioritized based on the presence of metabolic syndrome, diabetes status, and BMI. 50 patients were selected based on clinical judgment of overall medical need following assessment of the severity of obesity (BMI) and diabetes (HbA1c). Polypharmacy was a key factor in patient selection, with preference given to individuals on multiple medications particularly for conditions related to metabolic disease. Additionally, patients at risk of adverse events due to specific medications or combinations of medications were prioritized to ensure timely and effective intervention. Other factors also examined include the clinical profile of applicants including comorbidities, safety, and readiness to participate in the program. These criteria ensured that patients with the highest medical need and those at risk of complications were included promptly. Every employee was given access to the TOWARD smartphone app for education and engagement purposes and when clinical availability allowed further employees were enrolled. All patients were initially onboarded purely with clinical intent (details below).

### Statistics

Data was analyzed in both a per-protocol (PP) and an intent-to-treat (ITT) fashion (with the last observation timepoint being carried over).

Data management and statistical analyses were performed using R version 4.0.3 (with R Studio version: 1.3.1093), and all the R packages were updated to their latest version (30 April 2024). Descriptive data were summarized using mean and standard deviation and confidence intervals were calculated with mean bootstrap. Drug costs were estimated using those listed on GoodRx [GoodRx.com (accessed in January 2024)].

## Results

Data from 50 subjects were included in the intention-to-treat analysis. All subjects completed the first visit and nine subjects dropped out. Three of the subjects are no longer working for the company, one subject cited family reasons for dropping out, and the other dropouts did not give a reason for leaving the program (Figure 3). Of the 50 subjects, 23 (46%) were male and the median age at first visit was 52 (10). The mean starting weight was 123.8 kg (Table 1).

**Table 1:** Baseline characteristics and change in weight. *p < 0.05, **p < 0.01, ***p < 0.001.

	Baseline		1 Year		Change	
	Mean	SD	Mean	SD	Mean	SD
Age, years	50.2	9.7				
Weight, lbs. (kg)	270.7 (122.8)	51.0 (23.1)	227.7 (103.3)	43.9 (19.9)	−43.0 (19.5)***	25.2 (11.4)
BMI kg/m^2^	43.2	8.7	36.3	7.4	−6.9***	4.4

**Figure 4:**
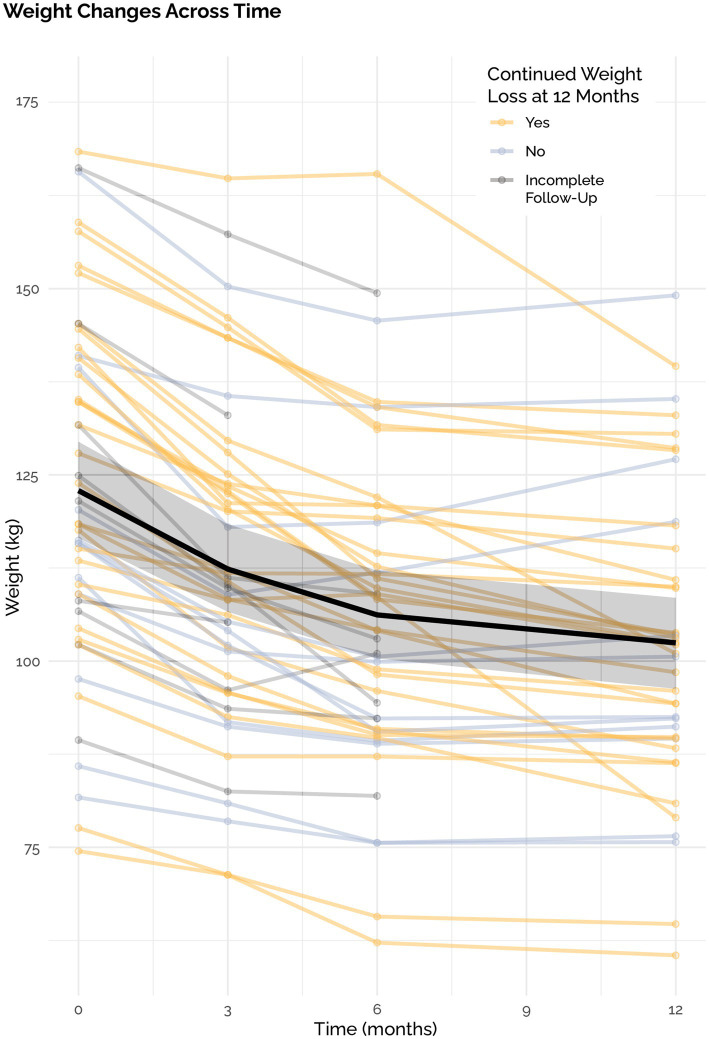
Weight loss across time. Confidence bounds are standard errors.

The mean weight loss was 19.5 ± 11.8 kg in the ITT analysis with a mean weight loss of 20.5 kg ± 11.8 in the PP analysis. This corresponded with an average weight loss of 15.5% (7.9%) of total body weight in the ITT analysis. 92% of individuals lost at least 5% of their body weight, 70% lost at least 10, 52% lost at least 15, and 30% lost >20%. All 50 out of 50 participants lost weight compared to their baseline. Of the 12 participants that did not continue to lose weight, mean weight loss was 12.3 kg. with a range of 1.6 to 23.2 kgs. Of the 50 participants, 38 continued to lose weight through 12 months and two stopped losing weight after month three and ten stopped losing weight after month six (Figure 4).

**Figure 5:**
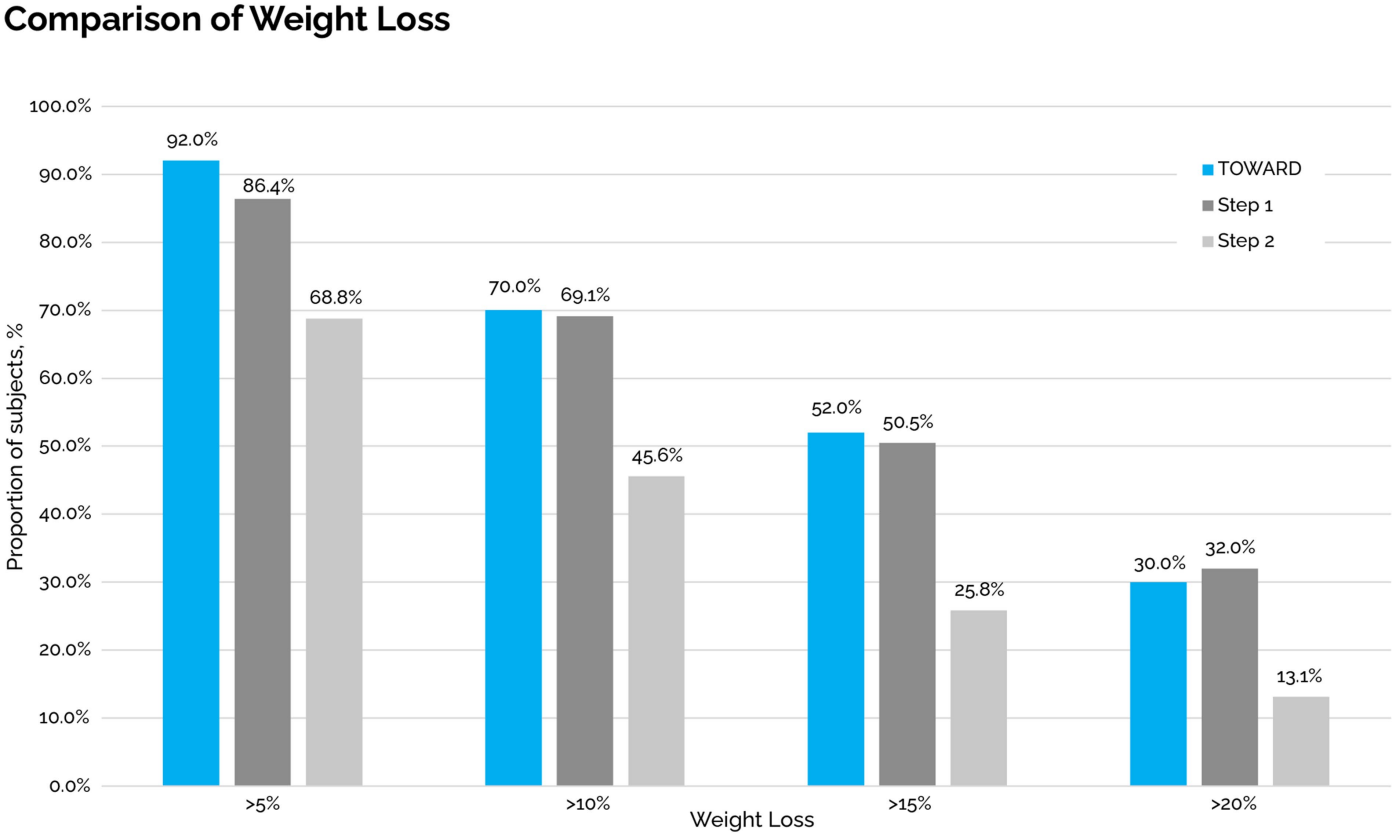
Weight loss threshold comparison to STEP 1 and STEP 2 trials.

**Figure 6:**
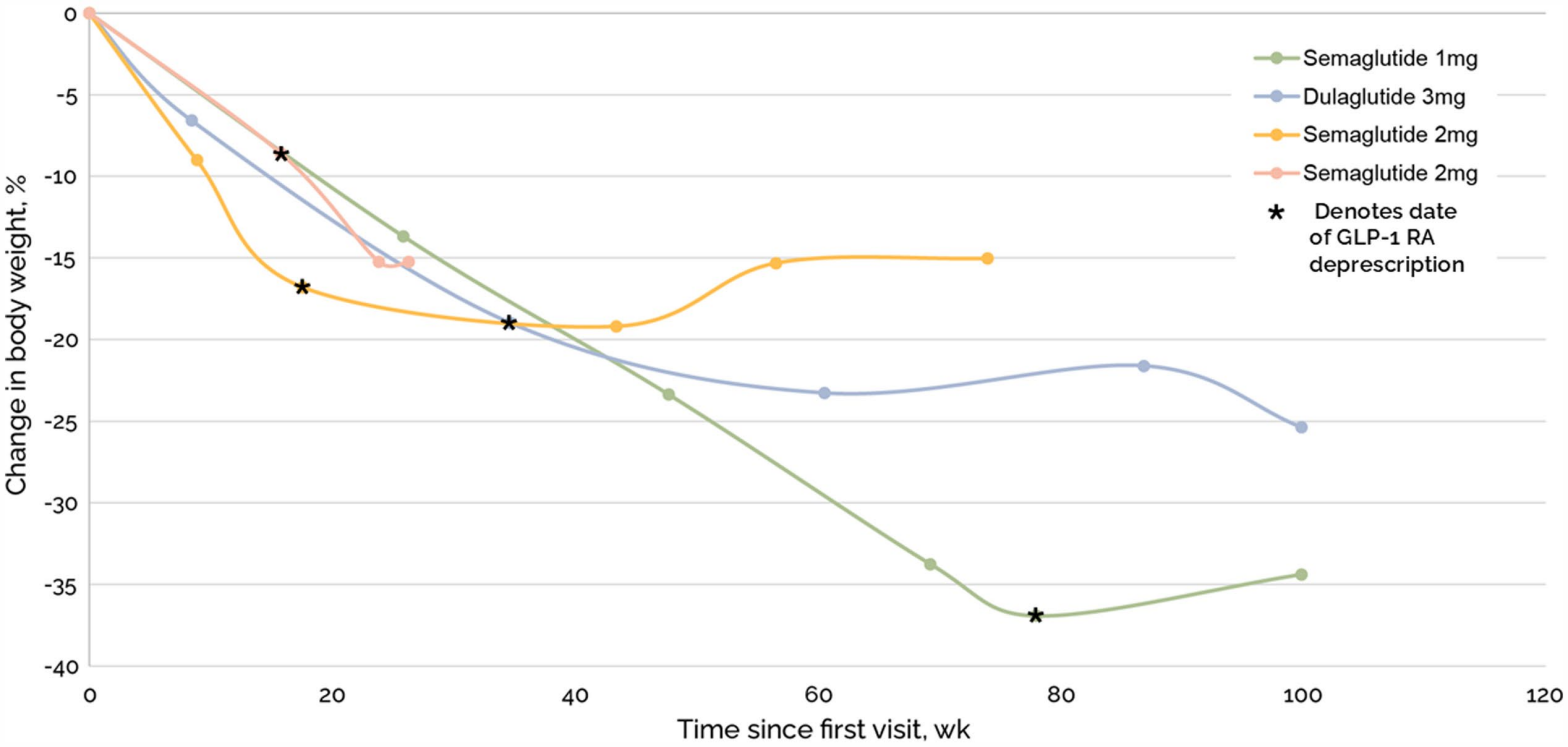
Continued weight loss of four participants after cessation of GLP-1RA.

Total weight loss was comparable in patients with and without GLP-1RA (Figure 5). Of the 96 medications that were deprescribed, four were GLP-1RA, including two semaglutide 2 mg, semaglutide 1 mg, and dulaglutide 3 mg. Mean time to last follow up after beginning taper and complete deprescription was 487 days and 270 days, respectively. One subject on semaglutide 2.0 mg stopped their medication without a taper. After complete cessation, subjects experienced a 4%, −8%, 2%, and − 7% change in weight (Figure 6).

A total of ninety six medications were deprescribed and eight medications were prescribed. Eighteen of these medications were for T2D or prediabetes, nine were for Gastroesophageal Reflux Disease (GERD), and 26 were for hypertension (Supplementary material). The eight medications added included amlodipine-olmesartan, two prescriptions of tirzepatide, levothyroxine, pravastatin and three prescriptions of rosuvastatin. One prescription of tirzepatide was added to help an individual who continued to score highly on the Yale Food Addiction Scale (YFAS) screening despite maintaining TCR for several months and another prescription of tirzepatide was started for persistently elevated HbA1c and history of amputations. Overall, direct cost savings from deprescription over the course of 1 year were approximately $111,740.00 from deprescription, whereas net cost savings over the year from medication changes was approximately $83,285.00, which annualizes to a savings of about $1,700.00 per subject (Supplementary material).

## Discussion

Our 1-year data of 50 subjects who underwent a TOWARD comprehensive metabolic clinic intervention demonstrated continued weight loss of −15.5% in an ITT analysis, with −16.6% weight loss in the PP analysis, and in the setting of medication deprescription, including GLP-1 RA drugs, with resultant cost savings. Our average weight loss was comparable to Semaglutide 2.4 mg in the STEP 1 trial which had an average − 14.9% weight loss from baseline at 68 weeks (5). However, as our sensitivity analysis showed, it is unlikely that this similarity is due to some of our patients using GLP-1RAs as these were only a small proportion of our cohort (8 out of 50 patients) and weight loss was comparable in patients with and without GLP1-RA. A main difference between our intervention and semaglutide 2.4 mg is cost; the annualized costs of semaglutide is roughly $13,000 while the TOWARD approach provided annualized cost savings of $1,700 through medication deprescription. We also reported four subjects who discontinued their GLP-1 RA with ongoing weight loss or < 5% weight regain.

These data add to a growing body of evidence that a multi-modal approach to lifestyle that includes TCR is a cost-effective strategy for improving metabolic health. In the UK, interventions that employ TCR have been shown to reduce costs associated with diabetes while demonstrating impressive clinical results with T2D remission (27). In companies with a large market capitalization, a telemedicine approach to TCR has demonstrated durable T2D remission along with cost-savings at 2 years (21). Given that only 6.8% of the adults in the United States have optimal cardiometabolic health (28), there is an urgent need to find sustainable, cost-effective methods like TOWARD to reverse the metabolic health epidemics in more diverse settings.

Critics of lifestyle interventions for weight loss and diabetes remission have suggested the lack of long-term durability secondary to adherence as a major limiting factor for persistent efficacy and weight loss maintenance (29). In the UK, a study demonstrated long term diabetes remission in 91 patients (27). In our present data, 76% continued to lose weight after 12 months in the ITT analysis. This suggests that intensive lifestyle interventions are viable and durable long-term options for chronic disease (27).

Limitations of this pilot study included absence of a formal control group, although we can and did compare our data to GLP-1RA published results at a similar time point. Further, the patients self-selected to apply for the program, which introduces bias to the results. The TOWARD intervention is also multimodal; thus, it is not possible to identify the relative contribution of each TOWARD component, heterogeneity of response to individual components throughout the cohort, and whether certain components synergized to improve metabolic health outcomes or adherence. The TOWARD intervention, being a lifestyle intervention, is also necessarily limited by present, widespread socioeconomic obstacles, including access to food, education, community, social norms, and metabolically informed healthcare supports. Thus, these pilot data, while promising, need to be taken in context of broader socioeconomic issues limiting efficacy of lifestyle interventions in the United States and elsewhere. Still, our results have many strengths, including potential for scalability given the nature of the intervention, relatively high rates of adherence despite participants not being educated on metabolic health at baseline, and a large effect size in terms of weight loss that is comparable to current presumed best practices (GLP-1RA), while deprescribing 96 medications, which resulted in medication cost savings for the employer.

Additional limitations include heterogenous data collection (which is true for any real-world clinical intervention). Given the nature of home, remote-monitoring and patient preference, many data points were heterogeneously collected. For example, due to technical limitations of the remotely-monitored home scale, inconsistencies arose caused by participants’ home-use, including issues such as wearing socks and shoes during measurements, making it impossible to ensure reliable and consistent body composition data across the cohort. As a result, we focused on body weight in this analysis. Lastly, although patients endorsed following a low-carbohydrate diet during clinic visits, the exact amount of carbohydrates eaten per day is unknown.

In conclusion, the TOWARD approach demonstrated robust improvements in weight and metabolic parameters with net deprescription of 88 medications across 50 patients. Additionally, there was significant cost savings through stopping medications that were no longer necessary due to lifestyle changes that were maintained for 1 year duration.

## Data Availability

The raw data supporting the conclusions of this article will be made available by the authors, without undue reservation.
