# Supplementary material for: TOWARD, a metabolic health intervention, demonstrates robust 1-year weight loss and cost-savings through deprescription
Source: Front Nutr. 2025 Feb 14;12:1548609. doi: 10.3389/fnut.2025.1548609 (PMC11868080; doi:10.3389/fnut.2025.1548609)
Supplement: Supplementary file 1 [file Table_1.PDF]

Supplemental Material. Detailed Deprescription Analyses

Prices are in USD and come from GoodRx® (or Amazon® if not Available)

	Medications	Dose	Monthly cost	Stopped	Savings per month	Added	Cost per Month
**Blood pressure**	*Amlodipine*	2.5mg daily	$ 14.00	1	$ 14.00		
	Amlodipine Olmesartan	5mg 20mg	$ 25.00		$ -	1	$ 25.00
	Chlorthalidone	25mg daily	$ 10.00	1	$ 10.00		$ -
	Hydrochlorothizide	12.5mg daily	$ 4.00	3	$ 12.00		$ -
	Hydrochlorothizide	25mg daily	$ 4.00	5	$ 20.00		$ -
	Benazepril	10mg daily	$ 10.00	1	$ 10.00		$ -
	Benazepril	20mg daily	$ 12.00	1	$ 12.00		$ -
	Valsartan	80mg daily	$ 20.00	1	$ 20.00		$ -
	Olmesartan	20mg daily	$ 20.00	1	$ 20.00		$ -
	Losartan	50mg daily	$ 10.00	2	$ 20.00		$ -
	Atenolol	25mg daily	$ 4.00	1	$ 4.00		$ -
	Amlodipine	5mg daily	$ 18.00	1	$ 18.00		$ -
	Amlodipine	10mg daily	$ 18.00	2	$ 36.00		$ -
	Lisinopril	10mg daily	$ 4.00	1	$ 4.00		$ -
	Lisinopril	30mg daily	$ 4.00		$ -		$ -
	Lisinopril	40mg daily	$ 5.00	1	$ 5.00		$ -
	Metoprolol tartrate	25mg bid	$ 10.00	1	$ 10.00		$ -
	Metoprolol ER	25mg daily	$ 10.00	1	$ 10.00		$ -
	Carvedilol	12.5mg bid	$ 18.00	0	$ -		$ -
	Spironolactone	100mg daily	$ 15.00	1	$ 15.00		$ -
	Furosemide	40mg daily	$ 5.98	1	$ 5.98		$ -
**Blood Sugar**	*Ozempic (Semaglutide)*	2mg weekly	$ 998.00	2	$ 1,996.00		$ -
	Semaglutide	1mg weekly	$ 998.00	1	$ 998.00		$ -
	Dulaglutide	3mg	$ 852.00	1	$ 852.00		$ -
	Dulaglutide	4.5mg	$ 852.00	1	$ 852.00		$ -
	Trulicity (Dulaglutide)	1.5mg	$ 850.00	0	$ -		$ -
	Sitagliptin	50mg daily	$ 1,023.00	1	$ 1,023.00		$ -
	Metformin	500mg daily	$ 10.00	1	$ 10.00		$ -
	Metformin	500mg bid	$ 14.00	1	$ 14.00		$ -
	Metformin	1000mg bid	$ 15.00	2	$ 30.00		$ -
	Metformin ER	1000mg daily	$ 17.00	3	$ 51.00		$ -
	Mounjaro (Tirzepatide)	15mg weekly	$ 1,136.00	0	$ -	2	$ 2,272.00
	Dapaglifozin	5mg	$ 474.00	1	$ 474.00		$ -
	Empaglifozin	10mg	$ 600.00	0	$ -		$ -
	Glipizide	5mg BID	$ 10.00	1	$ 10.00		$ -
	Linagliptin	5mg	$ 461.00	0	$ -		$ -
	Lantus solostar 40U	1200U monthly	$ 35.00	1	$ 35.00		$ -
	Lispro 75u daily	2,250u monthly	$ 51.50	1	$ 51.50		$ -
	Humulin R U-500 75u TID	6750U monthly	$ 1,032.00	1	$ 1,032.00		$ -
**Lipids**	*Atorvastatin*	5mg daily	$ 18.00	1	$ 18.00		$ -
	Atorvastatin	10mg daily	$ 18.00	2	$ 36.00		$ -
	Atorvastatin	20mg daily	$ 18.00	1	$ 18.00		$ -
	Atorvastatin	40mg daily	$ 18.00		$ -		$ -
	Ezetimibe	10mg daily	$ 8.00	1	$ 8.00		$ -
	Fenofibrate	150mg daily	$ 20.00	1	$ 20.00		$ -
	Gemfibrozil	600mg daily	$ 50.00	1	$ 50.00		$ -
	Cholestyramine	powder	$ 30.00	0	$ -		$ -
	Pravastatin	20mg daily	$ 19.00		$ -	1	$ 19.00
	Rosuvastatin	5mg daily	$ 15.00	1	$ 15.00	2	$ 30.00
	Rosuvastatin	10mg daily	$ 15.00		$ -	1	$ 15.00
	Rosuvastatin	20mg daily	$ 19.43	1	$ 19.43	0	$ -
	Baby aspirin		$ 3.59	2	$ 7.18		$ -
	Livalo (Pitavastatin)	4mg daily	$ 334.00	0	$ -		$ -
**GERD**	*Omeprazole*	20mg daily	$ 10.81	2	$ 21.62		$ -
	Omeprazole	40mg daily	$ 15.00	3	$ 45.00		$ -
	Pantoprazole	20mg daily	$ 18.77	1	$ 18.77		$ -
	Pantoprazole	40mg daily	$ 18.91	1	$ 18.91		$ -
	Famotidine	40mg daily	$ 8.00	1	$ 8.00		$ -
	Lansoprazole (prevacid)	30mg daily	$ 11.00	1	$ 11.00		$ -
**Mood**	*Bupropion*	150mg daily	$ 15.00	0	$ -		$ -
	Vilazodone (Viibryd)	40mg daily	$ 60.00	1	$ 60.00		$ -
	Trazodone	50mg daily	$ 10.00	1	$ 10.00		$ -
	Buspirone	10mg bid	$ 22.00	1	$ 22.00		$ -
	Xanax (alprazolam)	0.5mg daily	$ 11.00	1	$ 11.00		$ -
**Misc**	*Tamsulosin*	0.4mg daily	$ 10.00	1	$ 10.00		$ -
	Linaclotide	72mcg	$ 560.93	1	$ 560.93		$ -
	Dicyclomine	20mg daily	$ 8.76	1	$ 8.76		$ -
	Ergocalciferol	50000iu weekly	$ 5.00	2	$ 10.00		$ -
	Allopurinol	300mg daily	$ 14.50	1	$ 14.50		$ -
	Allopurinol	100mg daily	$ 7.00	1	$ 7.00		$ -
	Potassium Chloride	20meq daily	$ 10.00	1	$ 10.00		$ -
	Terbinafine	250mg daily	$ 30.00	1	$ 30.00		$ -
	Triamcinolone	430g jar	$ 14.00	1	$ 14.00		$ -
	Adderall	20mg BID	$ 31.40	1	$ 31.40		$ -
	Adderall	20mg daily	$ 20.00	2	$ 40.00		$ -
	Levothyroxine	50mcg daily	$ 10.00	0	$ -	1	$ 10.00
	Naltrexone	50mg daily	$ 30.00	1	$ 30.00		$ -
	Meloxicam	15mg daily	$ 28.00	1	$ 28.00		$ -
	Flonase	1 inhaler	$ 15.00	3	$ 45.00		$ -
	Amitriptylline	25mg daily	$ 10.00	1	$ 10.00		$ -
	Premarin	0.625mg daily	$ 200.00	1	$ 200.00		$ -
	Testosterone	200mg weekly	$ 23.00	2	$ 46.00		$ -
	Singulair	10mg daily	$ 15.00	1	$ 15.00		$ -
	Ondansetron	4mg daily	$ 19.50	1	$ 19.50		$ -
	Ondansetron	8mg	$ 33.00	1	$ 33.00		$ -
	Percocet	10mg tid	$ 33.00	1	$ 33.00		$ -
	Lunesta	3mg nightly	$ 20.00	1	$ 20.00		$ -
	Generlac	473ml solution	$ 17.00	1	$ 17.00		$ -
	Tolerodine ER	4mg daily	$ 34.00	0	$ -		$ -
	Liothyronine	5mcg daily	$ 13.00		$ -		$ -
	Estradiol	1mg daily	$ 10.00	0	$ -		$ -
	Gabapentin	300mg bid	$ 12.00	0	$ -		$ -
	Methocarbamol	500mg bid	$ 11.00	0	$ -		$ -
	Zolpidem	10mg nightly	$ 18.00	0	$ -		$ -
	Norethindrone	0.35mg daily	$ 12.00	0	$ -		$ -
				**Total saving per month**	$ 9,311.48	**Total cost per month**	$ 2,371.00
				**Total saving per year**	$ 111,737.76	**Total cost per year**	$ 28,452.00
				**Net savings: average per person**		1665.7152	
				**Total savings: average per person**		2234.7552	
